# Phagocytic response of astrocytes to damaged neighboring cells

**DOI:** 10.1371/journal.pone.0196153

**Published:** 2018-04-30

**Authors:** Nicole M. Wakida, Gladys Mae S. Cruz, Clarissa C. Ro, Emmanuel G. Moncada, Nima Khatibzadeh, Lisa A. Flanagan, Michael W. Berns

**Affiliations:** 1 Beckman Laser Institute and Medical Clinic, University of California, Irvine, Irvine, California, United States of America; 2 Department of Neurology, University of California, Irvine, Irvine, California, United States of America; 3 Sue & Bill Gross Stem Cell Research Center, University of California, Irvine, Irvine, California, United States of America; 4 Department of Anatomy and Neurobiology, University of California, Irvine, Irvine, California, United States of America; 5 Department of Biomedical Engineering, University of California, Irvine, Irvine, California, United States of America; 6 Department of Developmental and Cell Biology, School of Biological Sciences, University of California, Irvine, Irvine, California, United States of America; Hungarian Academy of Sciences, HUNGARY

## Abstract

This study aims to understand the phagocytic response of astrocytes to the injury of neurons or other astrocytes at the single cell level. Laser nanosurgery was used to damage individual cells in both primary mouse cortical astrocytes and an established astrocyte cell line. In both cases, the release of material/substances from laser-irradiated astrocytes or neurons induced a phagocytic response in near-by astrocytes. Propidium iodide stained DNA originating from irradiated cells was visible in vesicles of neighboring cells, confirming phagocytosis of material from damaged cortical cells. In the presence of an intracellular pH indicator dye, newly formed vesicles correspond to acidic pH fluorescence, thus suggesting lysosome bound degradation of cellular debris. Cells with shared membrane connections prior to laser damage had a significantly higher frequency of induced phagocytosis compared to isolated cells with no shared membrane. The increase in phagocytic response of cells with a shared membrane occurred regardless of the extent of shared membrane (a thin filopodial connection vs. a cell cluster with significant shared membrane). In addition to the presence (or lack) of a membrane connection, variation in phagocytic ability was also observed with differences in injury location within the cell and distance separating isolated astrocytes. These results demonstrate the ability of an astrocyte to respond to the damage of a single cell, be it another astrocyte, or a neuron. This single-cell level of analysis results in a better understanding of the role of astrocytes to maintain homeostasis in the CNS, particularly in the sensing and removal of debris in damaged or pathologic nervous tissue.

## Introduction

As the most numerous cells in the central nervous system (CNS), astrocytes serve an important role in maintaining homeostasis of the brain microenvironment. To maintain homeostasis, astrocytes provide structural support, aid in cell-to-cell communication, recycle neurotransmitters, and provide nutrients [1–3]. This has led to a greater realization of the importance of astrocytes in the CNS. However, a complete understanding of the functional role of astrocytes is lacking. In this study, we use high resolution imaging and selective single cell injury induced by laser nanosurgery to investigate the role of astrocytes in the phagocytosis of debris from dying and/or dead cells.

Neurons and astrocytes form interactive networks within the CNS. Disturbances of normal neuron-astrocyte interactions lead to neurodegeneration and progression of neurological diseases such as amytropic lateral sclerosis, Alzheimer’s, Huntington’s, and Parkinson’s disease [4]. In addition, there is a growing body of evidence for the role of astrocytes in detection, remodeling, and repair of nervous tissue following injury, such as in traumatic brain injury [5].

Through a process of reactive astrogliosis, astrocytes respond to varying amounts of brain injury and pathology in neurological disorders [1, 5]. During this process, astrocytes can limit damage to nervous tissue and aid in the restoration of normal function. Because of the importance of this process, a better understanding of the cellular and molecular basis of reactive astrogliosis is needed [3].

A study demonstrating that CNS astrocytes play a role in the damage-repair process showed astrocytes engulfing entire cell corpses in response to a non-discriminant “swath” of damage produced by moving a scalpel blade several times through a mixed-cell neural culture (6). These results suggested that astrocytes remove cell debris in order to protect surrounding healthy neurons from the toxic materials released by the dead and dying cells. Healthy cells were observed undergoing necrosis/cell death as a secondary injury phenomenon through contact with soluble mediators released from dead or dying cells [6]. Therefore, it is important to characterize astrocytes abilities to sense damage to surrounding cells and subsequently to phagocytose the damaged cell debris.

In this study, we utilize laser nanosurgery/nanoablation to induce catastrophic damage resulting in rapid cell death of a single astrocyte or neuron. The response of nearby un-irradiated astrocytes is to phagocytose either the damaged (dying) cell or the debris from rapid lysis of the irradiated cell. We characterize the cytological and behavioral changes of the responding astrocyte as it interacts with and eventually engulfs the damaged cell (or its debris), including the process of extensive endocytic vesicle formation. The system we describe permits the study of phagocytosis at the single cell level.

## Methods

### Cell culture

All applicable international, national, and/or institutional guidelines for the care and use of animals were followed. All animal housing conditions and dissection procedures were approved by and conducted according to the Institutional Animal Care and Use Committee (IACUC) at the University of California, Irvine. Mice were euthanized by CO_2_ asphyxiation, followed by cervical dislocation. A mixed population (astrocytes and neurons) of primary cells was isolated from the cerebral cortices of embryonic day 12.5 CD1 mice as previously described [7]. Cortical tissue was placed in dissection buffer containing 0.6% glucose in 1X DPBS with penicillin-streptomycin. Tissue was dissociated with 0.025% Trypsin-EDTA in PBS and trypsin neutralized with 100 mg/mL soybean trypsin inhibitor (Thermo Fisher). Isolated cells were plated onto Matrigel (Corning)-coated 35 mm glass bottom dishes (World Precision Instruments) at densities ranging from 50,000 to 100,000 cells per dish and cultured in MEM (Thermo Fisher) growth media supplemented with 2% horse serum, 5% fetal bovine serum, 100 ng/mL nerve growth factor (PeproTech), 0.5x B27 supplement, and 100 U/mL penicillin-streptomycin. A minimum of two days was allotted for cells to attach to the matrigel substrate prior to laser irradiation. Two days following dissection, media was replaced with MEM-based media supplemented with 2% horse serum, 100 ng/mL nerve growth factor (PeproTech), and 0.5x B27 supplement.

In addition to primary astrocytes, an established astrocyte type-I (Ast-1) line (clone CRL-2541) was received directly from ATCC. Ast-1 cells were carried in advanced DMEM media with 2% FBS, 1% glutamax, and 0.2% gentamicin/amphotericin B. Ast-1 cells were plated onto glass bottom 35mm imaging dishes, or imaging dishes coated for 1 hour in a 1:60 dilution of matrigel: DMEM.

### Immunostaining

Primary cultures were fixed with 4% paraformaldehyde for 10 minutes at room temperature. Cells were incubated overnight at 4°C with anti-GFAP antibody produced in rabbit (Sigma Aldrich), diluted in in 3% Triton-X and 5% bovine serum albumin in PBS. Following three wash repetitions with PBS, cells were incubated for 1 hour with Alexa fluor 488 conjugated goat anti rabbit antibody (Sigma) and a 1 minute incubation with Hoescht stain. Cells were imaged with epifluorescence following a final wash with PBS.

### Laser microscope

Two different Zeiss Axiovert inverted phase contrast microscopes, each coupled to a different laser system, were used. The back aperture of a Zeiss Plan-Apochromat 63x, 1.4 numerical aperture oil immersion objective was filled with the laser beam by expansion with a telescopic lens. The resulting focused laser spot at the microscope image plane was near-diffraction-limits, approximately 0.8 μm for the near-infrared 800 nm femtosecond laser and 0.5 μm for the 532 nm nanosecond laser. The laser power in the focal spot was controlled by a circular polarizer mounted on a motorized rotational stage. A Uniblitz electromechanical shutter was computer controlled to allow a range of 10–50 ms burst of laser pulses into the microscope system. Specifications of the femtosecond laser microscope system have been described previously [7]. Position of the beam in the focal plane was controlled using a motorized XY fast scanning mirror in the beam path. Microscope images were captured with a Hamamatsu Orca-R2 CCD camera. The custom developed Robolase computer program on a Labview platform was used to control settings of the laser microscope system including power, exposure time, and position of the focused laser beam. Robolase also controlled camera settings including exposure time, image acquisition, light source, and filter set.

In the present study, two short pulsed laser systems were used: (1) a Coherent Mira 900 Ti:Sapphire femtosecond laser operating at 800 nm wavelength with pulse repetition rate of 76 MHz, and (2) a Coherent Prisma 532 nm second harmonic Nd:YVO_4_ 18 nanosecond laser operating at 20 KHz. The femtosecond laser was utilized at an average optical intensity of 3.4x10^8^ W/m^2^ at the focal point. The nanosecond laser was utilized at an average optical intensity of 4.5x10^8^ W/m^2^ at the focal point. Lethal cell damage/cell lysis was accomplished by irradiating either the nucleus or the cytoplasm of the target cell. Both lasers were exposed to cells within a single z-axis plane, confining damage to 0.5 to 0.8 μm (532nm and 800 nm lasers, respectively). The average cortical astrocyte was 5–7 μm thick (as determined by confocal microscopy), thus laser damage is confined to approximately 10% of the thickness of an astrocyte. Cells were observed by phase contrast microscopy for at least 1 hour following laser irradiation. When utilized at high powers, both the nanosecond and the femtosecond lasers killed cells rapidly upon exposure. Therefore, the data of all the astrocyte studies from both laser systems were combined.

Cells on the laser microscope stage were maintained at 37°C, 50% humidity and 5% CO_2_ using an Ibidi on-stage heating and incubation unit systems (Ibidi Inc).

### Live/Dead cell analysis

Live versus dead cell analysis was performed using the propidium iodide (PI) (ThermoFisher) exclusion assay. Cells were grown in phenol-red-free DMEM media (for SILAC) (ThermoFisher) supplemented with 2% fetal bovine serum, 3151 mg/L glucose, 147.5 mg/L L-arginine HCL, and 91.25 mg/L L-lysine HCL. Prior to laser exposure, PI (0.5 μM) was added to the media. Fluorescence imaging was performed on a Zeiss Axiovert using a Cy3 filter set.

### Vesicle pH analysis

Detection of acidic environments of vesicles was accomplished utilizing pHrodo Green AM Intracellular pH indicator (ThermoFisher Scientific). Cells were grown in phenol-red-free DMEM media (for SILAC) (ThermoFisher) supplemented with 2% fetal bovine serum, 3151 mg/L glucose, 147.5 mg/L L-arginine HCL, and 91.25 mg/L L-lysine HCL. Prior to laser exposure, cells were incubated for 30 minutes in 5 μM pHrodo with 1000x powerload concentrate at 37°C. Fluorescence imaging was performed on a Zeiss Axiovert using a GFP filter set.

### Quantitation and statistical analysis

Phagocytosis of the debris from dead cells was determined by analysis of digital time-lapse movies. Distances between cells were determined by measurements of individual digital images from the time-lapse movies using Image J (NIH). Statistical analysis comparing experimental values utilized the two proportion Chi square test to determine the 2 tailed p values. When only two samples were compared, t-tests were used to determine 2 tailed p values (denoted in results text).

## Results

### Astrocyte identity

**Fig 1:**
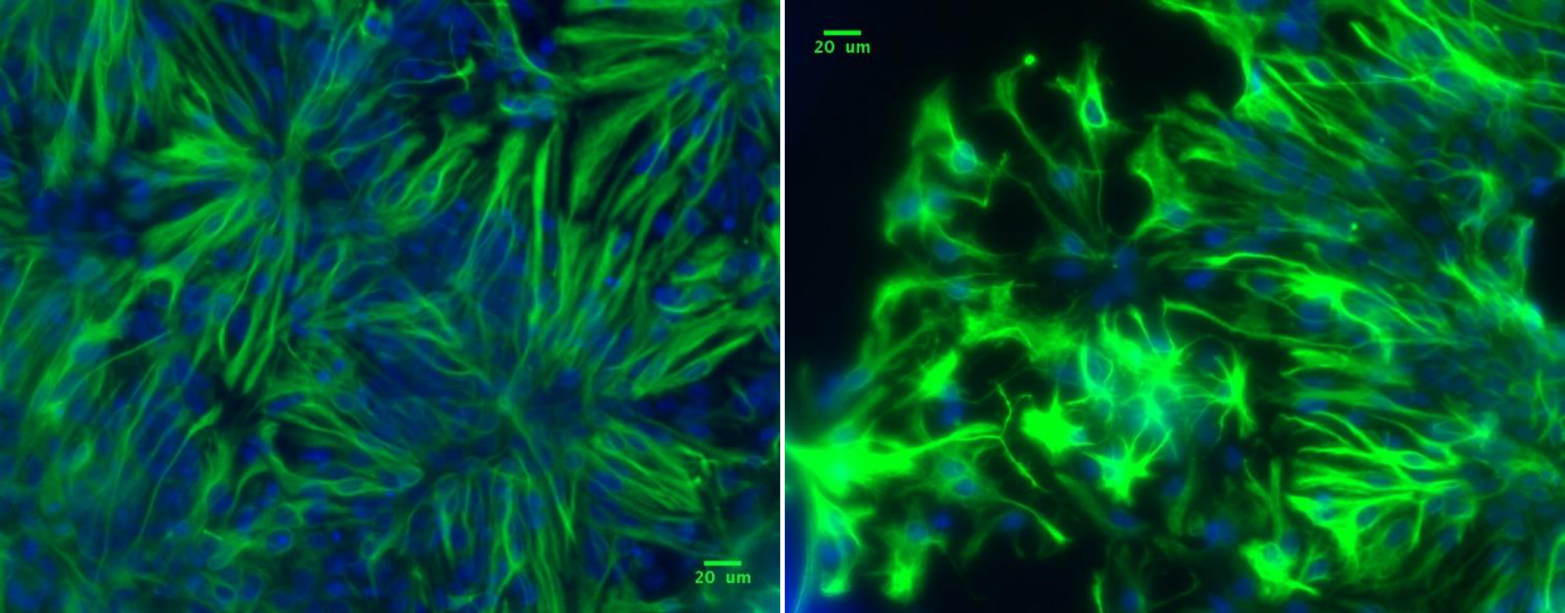
Immunostaining of cortical astrocytes with GFAP (green) and Hoesct (blue) reveals 95% of cells positively express GFAP.

To confirm that a majority of cells derived from the cortical culture were astrocytes, cultures were analyzed for the expression of astrocyte marker glial fibrillary acidic protein (GFAP) through antibody staining. GFAP immunostained cultures confirms that 95% of cells present are GFAP positive astrocytes (Fig 1).

### Laser induced astrocyte cell death

**Fig 2:**
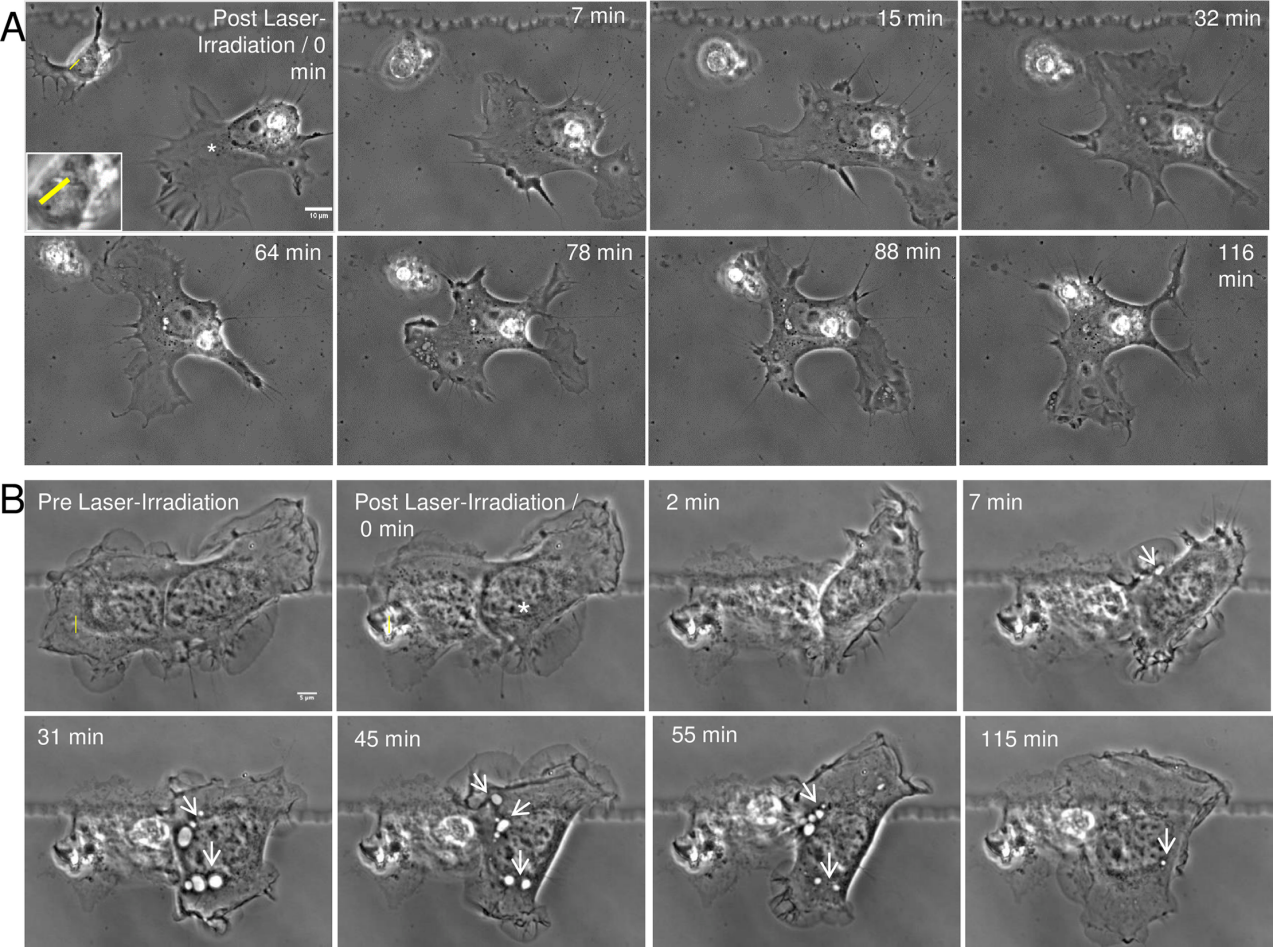
Neighboring astrocyte initiates phagocytosis of irradiated damaged/dead cell. **A.** One of two single, isolated astrocytes (not sharing a membrane connection) from a cortical culture is lysed via laser nanosurgery. The “Post Laser-Irradiation / 0 min” image shows the two astrocytes immediately following laser exposure. The laser exposure site is denoted by a yellow ROI (magnified image of laser-targeted region visible in inset). Seven minutes following laser irradiation, necrosis of the targeted astrocyte is observed. The responding astrocyte (white asterisk) approaches the lysed cell material at 7 to 15 minutes, and by 32 minutes establishes a membrane connection. In the 0 minute image, the leading edge of the responding astrocyte is oriented toward the bottom of the image. Following laser irradiation, the responding astrocyte retracts its leading edge and subsequently migrates toward the necrotic laser-irradiated cell. Prior to and immediately following laser irradiation, there are no obvious vesicles in the cytoplasm of the leading edge of the responding astrocyte. However, seven minutes following laser irradiation, vesicles begin to form at the cell periphery and are observed migrating toward the nucleus. By 116 minutes, the responding astrocyte engulfs the necrotic material via phagocytosis. B. A representative astrocyte type-I (Ast-1) cell from an immortalized cell line responded to laser irradiation of a neighboring astrocyte (yellow ROI). Prior to laser exposure, the responding astrocyte (white asterisk) shared a membrane connection to the laser irradiated cell. Phagocytosis of the irradiated/killed cell was initiated seven minutes following laser exposure. An increase in membrane ruffling and extension toward the damaged cell or its debris was observed. Endocytic vesicles moved from the cell periphery toward the nucleus (white arrowheads). Vesicles increased in size and number over the initial 45 minutes, and appeared to break down 55–115 minutes following laser irradiation.

**Fig 3:**
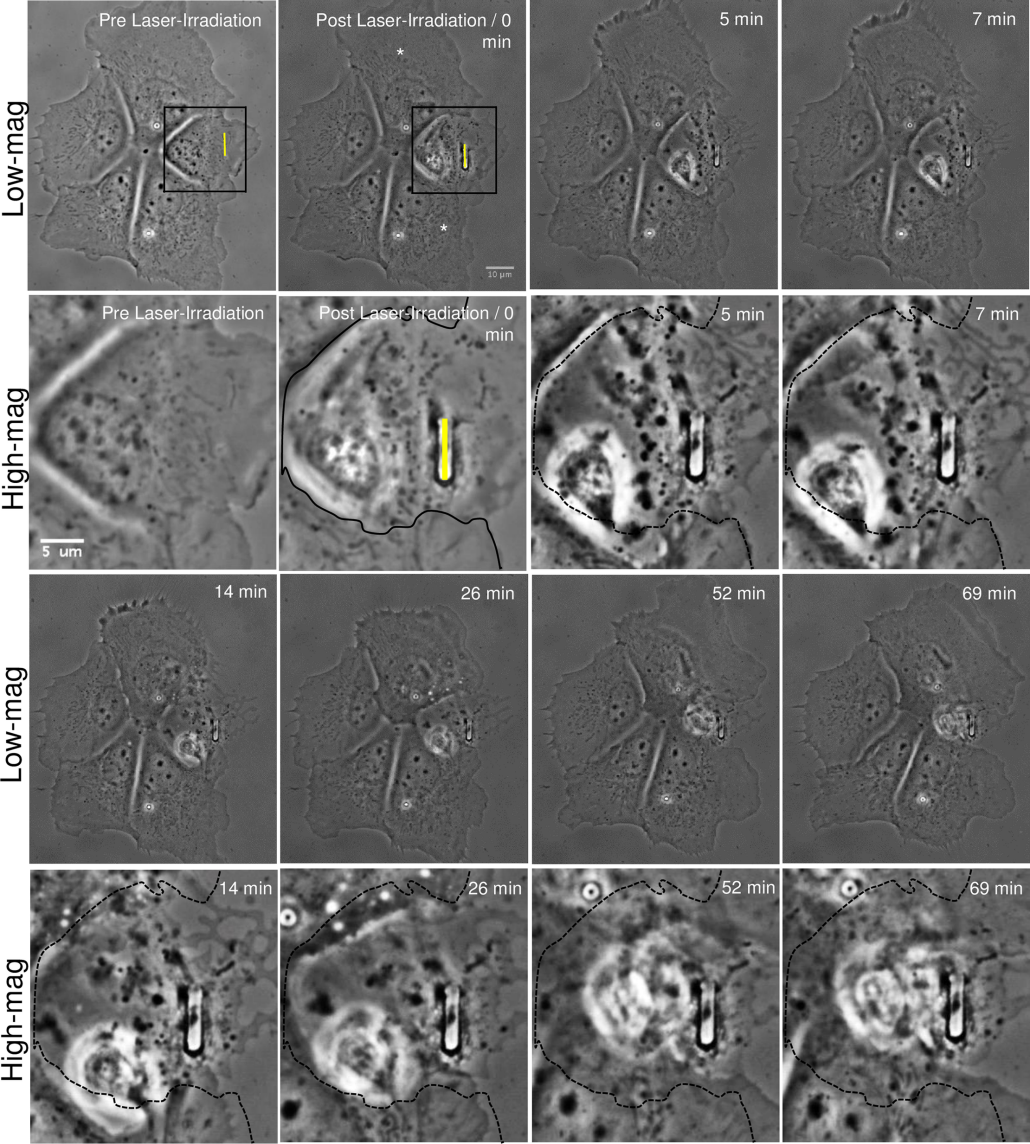
Phagocytosis of a laser-irradiated astrocyte within a cell cluster. An astrocyte within a five-cell cluster is lysed via laser nanoablation along the yellow ROI, visible in the first two images. Contrast enhanced, higher magnification images of the irradiated region are displayed in the second and fourth rows labeled “High-mag”. High magnification images correspond to the region within the outlined box in both pre and post laser-irradiated cell images. Immediately following laser exposure, the targeted cell shows visible signs of death including membrane retraction from the dish surface, phase paling of the nucleus, and phase dark material aggregating around the laser-irradiated site. Two cells closest to the targeted cell (white asterisks) share significant membrane contact with the irradiated cell. Between 5–69 minutes, the two astrocytes respond by extending their membranes toward the dead cell debris that they eventually surround and engulf. To better define the advancement of the responding astrocytes’ lamellae into the region of the irradiated cell, the boundary edge of the irradiated cell is traced with a solid black line in the post laser-irradiated region. This line is represented as a dashed black line in the subsequent high-magnification images. As the lamellae advances, vesicles form within the two responding cells starting 14 minutes following laser irradiation. The two other cells within the cluster, not in close proximity (and not sharing extensive membrane contact with the irradiated cell), do not exhibit any visible response following laser irradiation of the targeted cell.

**Fig 4:**
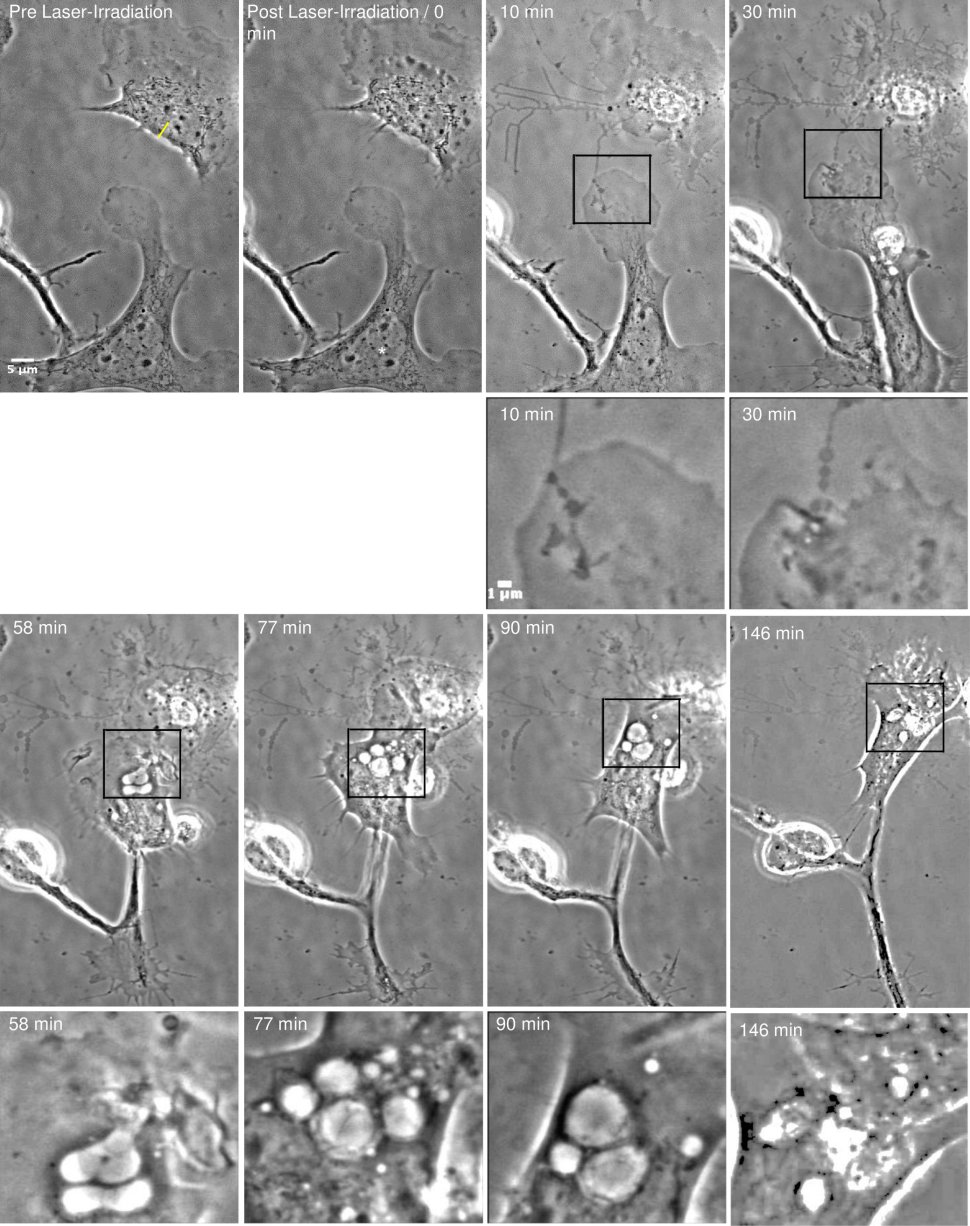
Vesicle formation in responding astrocytes. The astrocyte located in the top right in the Pre Laser-Irradiation image was targeted with the laser along the ROI indicated by the yellow line. Irradiation results in retraction of the cell body and thickening of filamentous processes that physically link the astrocyte with the responding cell (white asterisk). These filaments are noticeably more prominent 10 minutes following laser irradiation. After laser irradiation, the leading edge of the responding astrocyte approaches the lysed cell. Black outline-boxes correspond to the position of the magnified images displayed in image rows two and four. The responding astrocyte, establishes a substantial membrane connection with the cell debris 10 minutes following laser irradiation. At 30 minutes following irradiation, vesicles begin to form at the cell periphery closest to the cell debris. By 58 minutes post-laser, large vesicles have formed in the responding astrocyte, and continue to form over a 146 minute period. The responding astrocyte continues to advance on the cellular debris, with a continuous formation of large endocytic vesicles. By the end of the observation period, minimal cell debris remains of the irradiated cell.

To examine whether laser nanoablation/nanosurgery could be utilized to induce rapid cell death of targeted mammalian cortical brain cells, single primary mouse astrocytes were irradiated with either the femtosecond or nanosecond short-pulsed lasers. Both lasers accurately target cells for laser nanosurgery [8–10]. The laser was focused into the nucleus or cytoplasm of the targeted cell, and subsequently scanned over a region of interest (ROI) defined by Robolase software. Time-lapse imaging revealed that within seconds following laser exposure, dramatic changes to the nucleus and membrane of the irradiated cell were observed, including cytoplasmic blebbing and formation of multiple cytoplasmic phase-dark spots (Figs 2–4 Post Laser-Irradiation). Expulsion of cellular material into the surrounding extracellular space occurred soon after laser irradiation. PI treatment showed enhanced fluorescence emission of the laser-irradiated cell, confirming that it was killed. (S1 Fig) Membranes of neighboring cells remained intact following laser exposure of the target cell, and displayed no change in PI fluorescence. This suggests that laser damage was confined to the targeted cell with no collateral damage to the neighboring cells (S1 Fig). The visual morphological changes observed by phase contrast microscopy and PI staining were consistent with rapid necrosis and cell death following laser exposure.

### Astrocyte phagocytosis

To determine the ability of astrocytes to phagocytose laser-irradiated necrotic cells, time-lapse microscopy was used to observe cell-to-cell interactions. Cells were observed for a minimum of one hour post-irradiation to determine if the injury was detected by neighboring astrocytes (Fig 2, S1 Fig). The targeted cell is labeled with a yellow ROI and the neighboring, responding cells are labeled with a white asterisk (*). In neighboring astrocytes, dramatic changes in cell morphology were observed, including increased amounts of membrane ruffling and the formation of filopodia projecting toward the irradiated cell or its debris. In 104/239 cases (44%), neighboring astrocytes migrated toward necrotic cells and engulfed the cellular debris. Note the decrease in the distance between the astrocyte and the cell debris (Fig 2A). At the membrane region where the responding astrocyte establishes physical contact with the cellular debris, an increase in membrane activity (ruffling) was observed (Fig 2A, 32 minutes, S2 Fig; Fig 2B 7 min). Ruffling consistently resulted in the formation of endocytic vesicles within the responding astrocyte, as depicted by white arrows in Fig 2B. In addition, junctions between the cell and debris formed, and were maintained during the observation period; some maintaining contact for several hours after laser irradiation. In 83/104 cases (80%), primary astrocytes remained connected to the debris for the remainder of the observation period. However, occasionally the astrocyte un-coupled from the debris and reattached after several minutes (S2 Fig). The cellular interaction shown in Fig 2 continued for the 116 minutes observation period. Reactive astrocytes (both primary and immortalized) are typical examples of the dramatic cytoplasmic migration toward, and subsequent phagocytosis of the debris of laser-irradiated cells.

To determine if single, isolated cells respond differently than aggregates of cells, we targeted individual cells in a multicellular astrocyte cluster where each cell shared significant membrane attachments with neighboring astrocytes (Fig 3, S3 Fig). The two asterisk-labeled cells in Fig 3 extended lamellae toward, and surrounded the lysed cell. Rows 2 and 4 of Fig 3 correspond to magnified images of the region denoted within the black outlined-box. The leading edge advanced into the region of the irradiated cell, indicated by membrane processes extending beyond the black line that corresponds to the cell border at the time of laser-irradiation. Responding cells continued to interact with the cell debris until minimal debris was visible. This process resulted in dramatic changes in cell shape and position relative to remaining cells. By the end of the 70 minute observation period, adjacent cells appeared to have replaced the laser targeted cell. Other than a slight change in cell shape, no further changes in the other two cells (that do not share membranous connections with the targeted cell,) within the cluster were observed. No vesicle formation or blebbing was observed in cells with no membrane contact with the irradiated cell. These non-responding cells were separated from the ROI at a minimum distance of 25–28 μm. This is different from the responding phagocytic astrocytes that exhibited significant visible vesicle formation. Primary astrocytes connected via membrane to laser-damaged/killed astrocytes engulfed the irradiated cell or its debris in 71/111 cases (64%). Thus, both isolated single cells as well as cells in a cluster responded by phagocytosis of cell debris.

### Vesicle formation in responding astrocyte

The formation of vesicles was observed in responding astrocytes after the cell established a membrane connection with the damaged/dead cell. The responding astrocyte in Fig 4, establishes a substantial membrane connection with the irradiated cell. Initially, multiple small vesicles formed at the cell periphery in contact with the necrotic cell body (Fig 4, 58 minutes Post Laser-Irradiation). Vesicles are clearly visible in high-magnification images (Fig 4, second and fourth rows). The high-magnification images correspond to the region outlined in black in the low-magnification images. Within minutes of the initial contact of the astrocyte cell membrane with the dead cell (or its debris), vesicles increased in size and moved toward the interior of the responding astrocyte. Vesicle diameters were as large as 6 μm in width and 9 μm in length (Fig 4, 90 minutes Post Laser-Irradiation). The largest area the vesicle in Fig 4 developed into was 27.4 μm^2^. This astrocyte continues to engulf cellular debris over a 146 minute observation period. As the cell advances toward the cellular debris, additional vesicles continue to form and break down. A majority of the cellular debris is removed over the observation time, resulting in almost no visible cell debris at 146 minutes post irradiation. (S4 Fig)

### Astrocyte phagocytosis of damaged neurons

**Fig 5:**
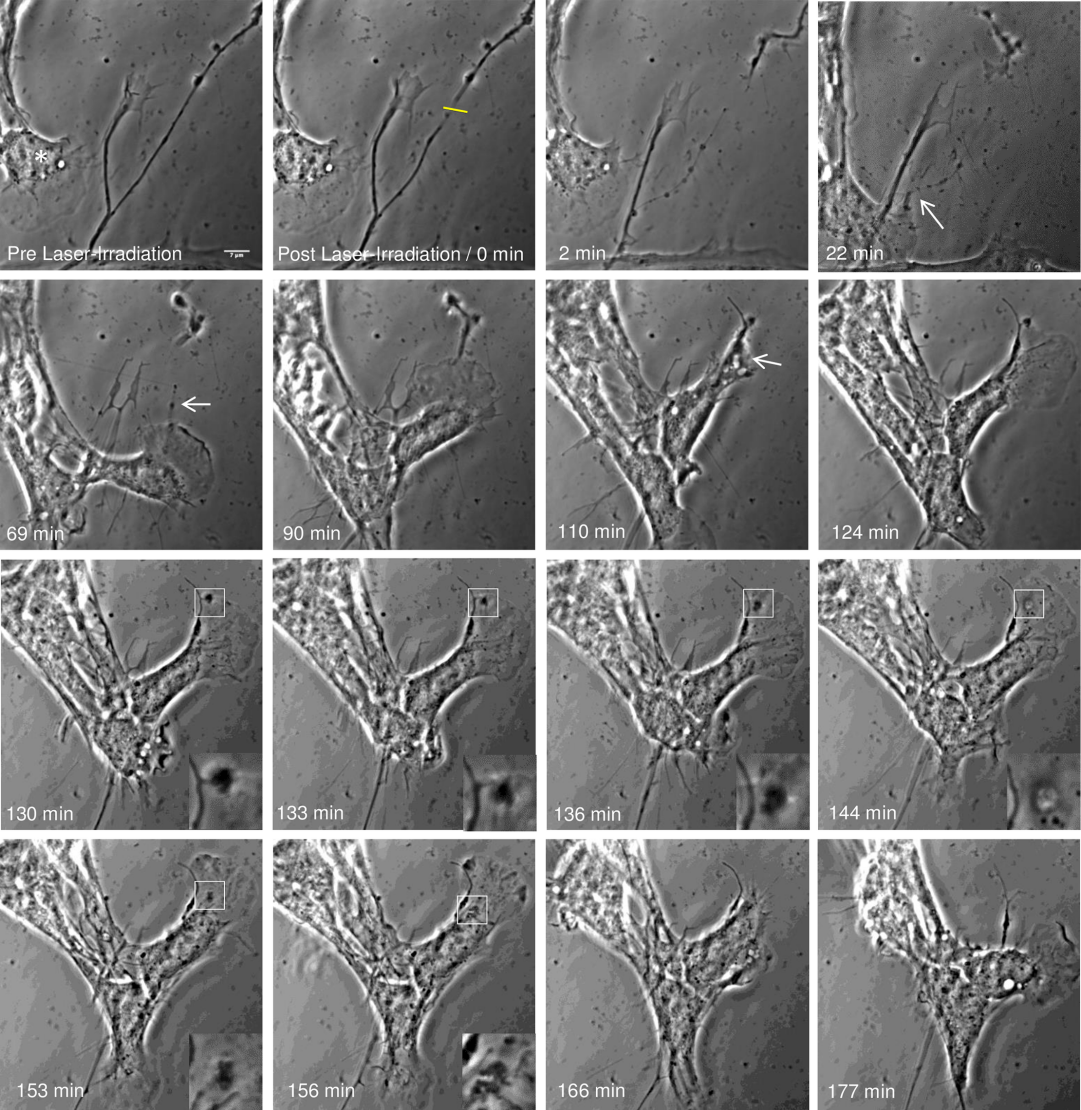
Astrocyte phagocytosis of dissected neurite debris. Phase contrast images (contrast enhanced) depicts the uptake of a neuron process by an astrocyte residing to the left of the process. Laser exposure to an axon (yellow ROI) results in thinning, blebbing, and full dissection of the growth cone. Following the dissection, the detached growth cone continues to actively move. At 22 minutes post laser irradiation, the neighboring astrocyte physically connects to the damaged process and begins to pull on debris (white arrow). The responding astrocyte (asterisk) continues to move along the damaged neurite between 57 and 69 minutes post laser irradiation in the direction of the growth cone. The astrocyte reaches the active growth cone 90 minutes following laser irradiation. Magnified insets highlight the intake of the growth cone, including phase dark material at the periphery of the cell membrane, engulfment of the debris, formation of endocytic vesicle, and movement of the debris toward the cell center. 124 min following laser irradiation, the leading edge is reformed followed by cell migration in the opposing direction, revealing no debris from growth cone remaining in the previously occupied region.

To better understand the amount of cellular damage required to stimulate the phagocytic response of astrocytes, microirradiation was used to sever individual axons within the vicinity of neighboring astrocytes. Fig 5 and S5 Fig demonstrates the full dissection of a growth cone from the axon of a neuron. During this time, a neighboring astrocyte responds by migrating toward the damaged region of the neuron, and establishing local contact with the damaged process 22 minutes post laser irradiation (arrow). The responding astrocyte continues to migrate along the damaged process and toward the growth cone making local contact 90 minutes post laser irradiation. Once a physical connection is established between the plasma membrane of the astrocyte and the previously severed growth cone, the responding astrocyte engulfs the remaining growth cone and subsequently forms endocytic vesicles. Magnified insets (lower right corners between 130 and 156 minutes post laser irradiation) of this process highlights the engulfment of the growth cone fragments and subsequent formation of the endocytic vesicle. Once the debris is engulfed within the cell, images track the movement of phase dark debris toward the cell center (magnified insets) between 153 and 156 minutes post laser irradiation. Following phagocytosis of the debris, the cell migrates in the opposite direction, revealing no cell debris remaining in the region previously occupied by the growth cone (166 and 177 minutes post laser irradiation).

### Responding cells observed to form endocytic vesicles are GFAP positive

**Fig 6:**
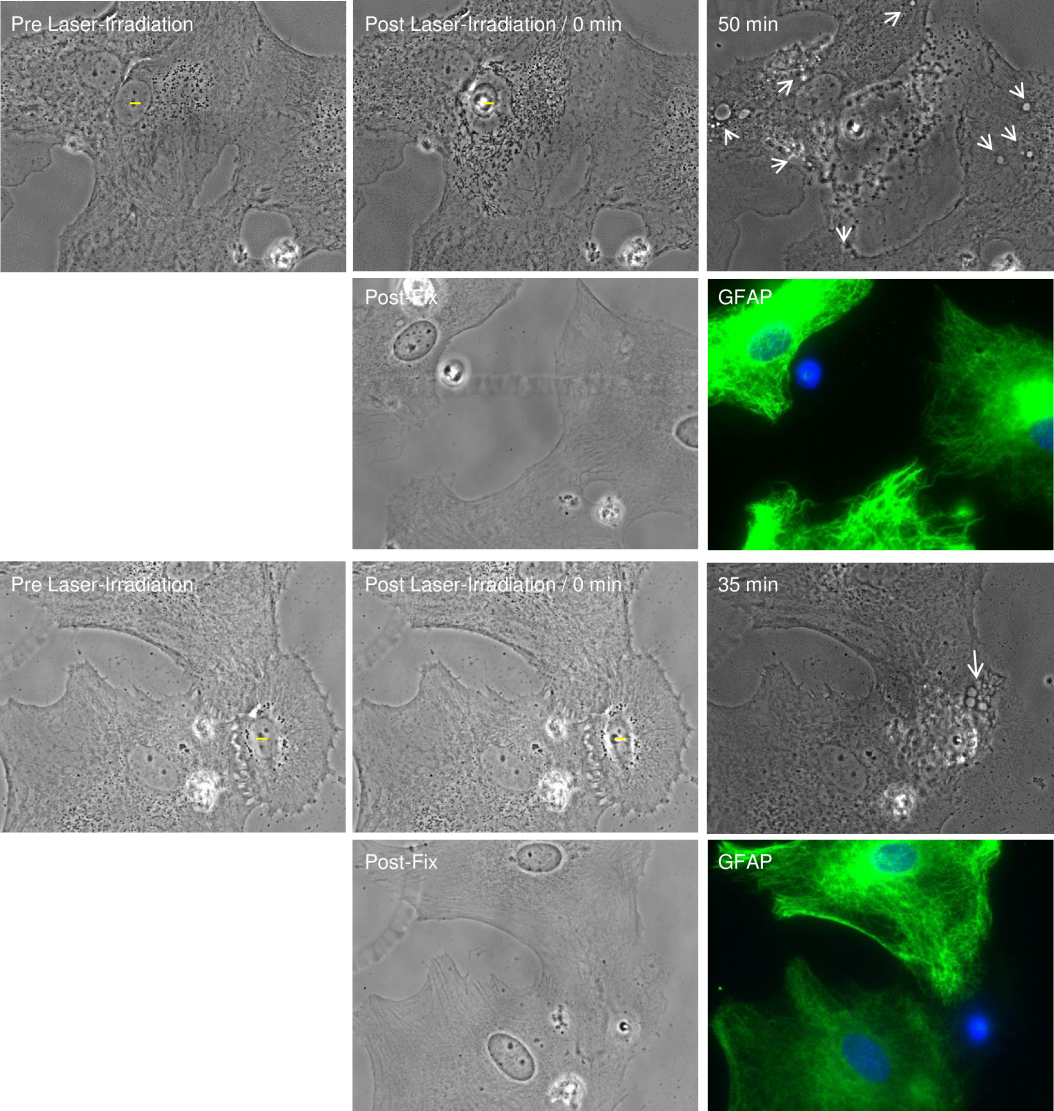
Responding astrocytes that form vesicles stain positive for GFAP. Observation of two sets of cells forming endocytic vesicles, highlighted by white arrows (50 minutes and 30 minutes post laser-irradiation, respectively) is displayed in rows 1 and 3. Phase contrast and corresponding immunofluorescence image following fixation and staining of previously observed cells displayed in rows 2 and 4, labeled “Post-Fix” and “GFAP”. “GFAP” immunofluorescence images are a composite of GFAP signal in green and DNA/Hoechst stain in blue.

To confirm the identity of responding cells, cells that formed endocytic vesicles were fixed, stained for GFAP, and imaged. Fig 6 demonstrates the progression of two sets of cells before laser-irradiation, after laser-irradiation, and 50 and 35 minutes, respectively, following laser irradiation. Following laser irradiation, cells adjacent to laser-targeted cells formed multiple endocytic vesicles, visible at white arrows in phase contrast images (rows one and three of Fig 6). After fixation and immunofluorescence staining, observed cells were relocated and imaged. Fluorescence GFAP images, in rows two and four of Fig 6, confirms that responding cells are display an intense fibrillar GFAP pattern, as visualized in green. Hoescht staining of DNA is visible in blue in composite fluorescence images. In both examples, residual DNA of the laser-irradiated cell remains. Staining of individual cells observed to form endocytic vesicles confirms responding cells are GFAP positive astrocytes.

### Propidium iodide in endocytic vesicles of responding astrocytes

**Fig 7:**
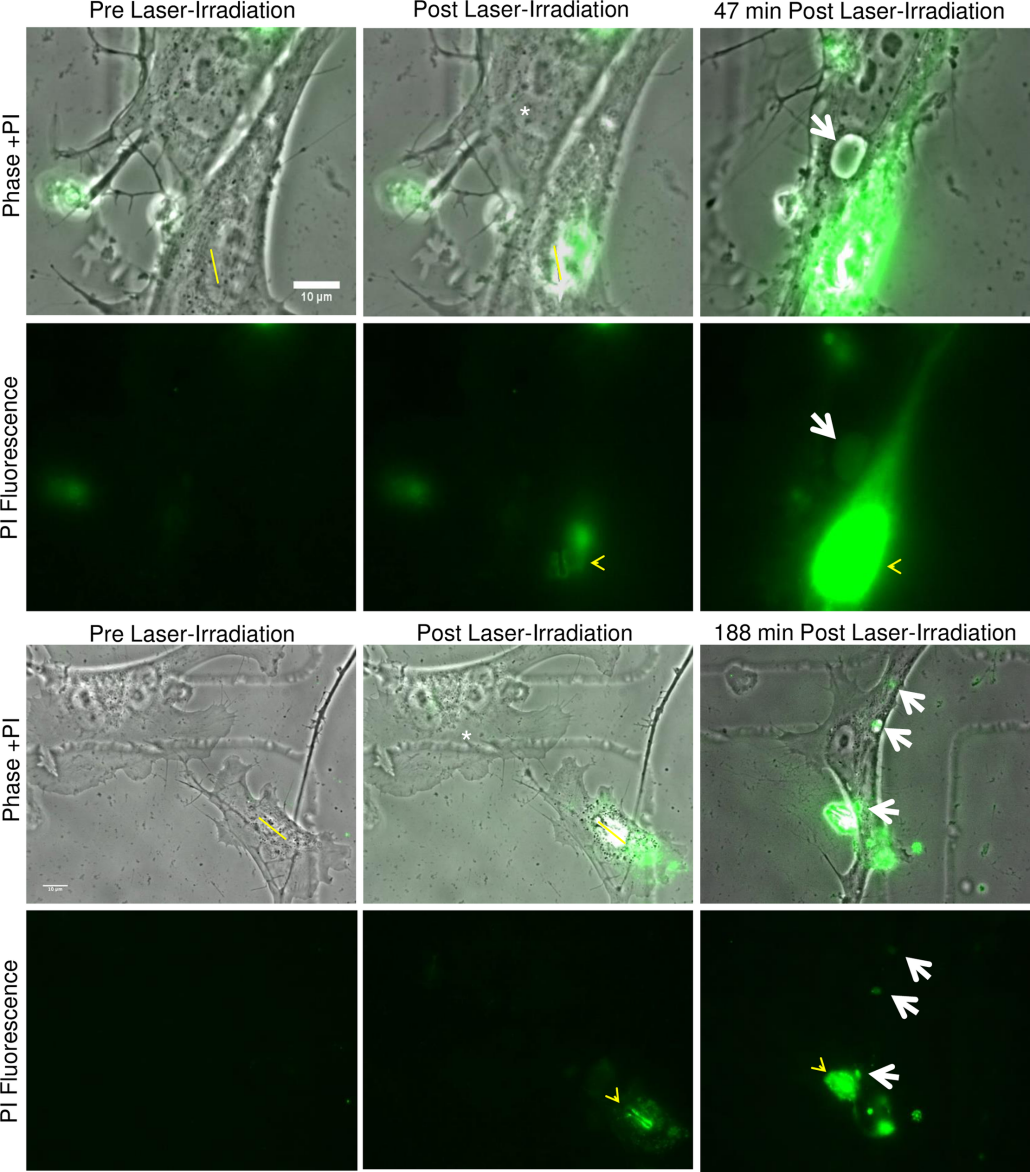
Propidium iodide stained nuclear material is incorporated into endocytic vesicles of responding astrocytes. Prior to laser exposure, PI was added to the medium surrounding primary astrocytes. Minimal intracellular PI fluorescence was observed within living cells, as visualized in the “Pre Laser-Irradiation, PI Fluorescence” images. After laser irradiation, PI is incorporated into the exposed DNA of the targeted cell along the yellow ROI, and was visualized as an increase in fluorescence intensity in “Post Laser-Irradiation” images (yellow arrow heads). Fluorescence of the exposed nuclear material continues to increase, producing a bright signal in the region of the targeted nucleus. This was visible 47 minutes post fluorescence in the first example and 188 minutes in the second example. Large endocytic vesicles were observed within the responding astrocytes (white asterisk) 47 and 188 minutes following laser-irradiation. Overlay images of phase and fluorescence confirm that the PI-stained nuclear material was located within the large endocytic vesicles (white arrows).

To verify that vesicles formed within the responding astrocyte contained cellular debris from the laser-targeted/killed cell, propidium iodide (PI) was added to the culture media of astrocytes prior to laser irradiation. Prior to laser exposure, no intracellular PI fluorescence was observed within living cells (Fig 7 PI Fluorescence Pre Laser-Irradiation). Laser exposure to either the nucleus or cytoplasm of individual astrocytes resulted in rapid cell death and the incorporation of PI. (ROI shown in yellow in Fig 7 Pre and Post Laser-Irradiation Phase +PI) Fluorescence was brightest along the targeted ROI (yellow arrowhead, PI Fluorescence Post Laser-Irradiation). PI staining of both cytoplasm and the nucleus of irradiated cells confirmed cell death following laser exposure (S6 Fig).

During the astrocyte phagocytic response, some of the large vesicles incorporated PI-stained nuclear material from the killed cell (white arrows in Fig 7). Overlay of phase contrast and PI fluorescence images confirm co-localization of PI-incorporated nuclear material and the endocytic vesicle within the responding astrocyte (see the two examples in Fig 7). PI-labeled nuclear material was detected in newly formed vesicles at 47 and 188 min post irradiation in the two responding astrocytes.

### Acidic endocytic vesicle formation in responding astrocytes

**Fig 8:**
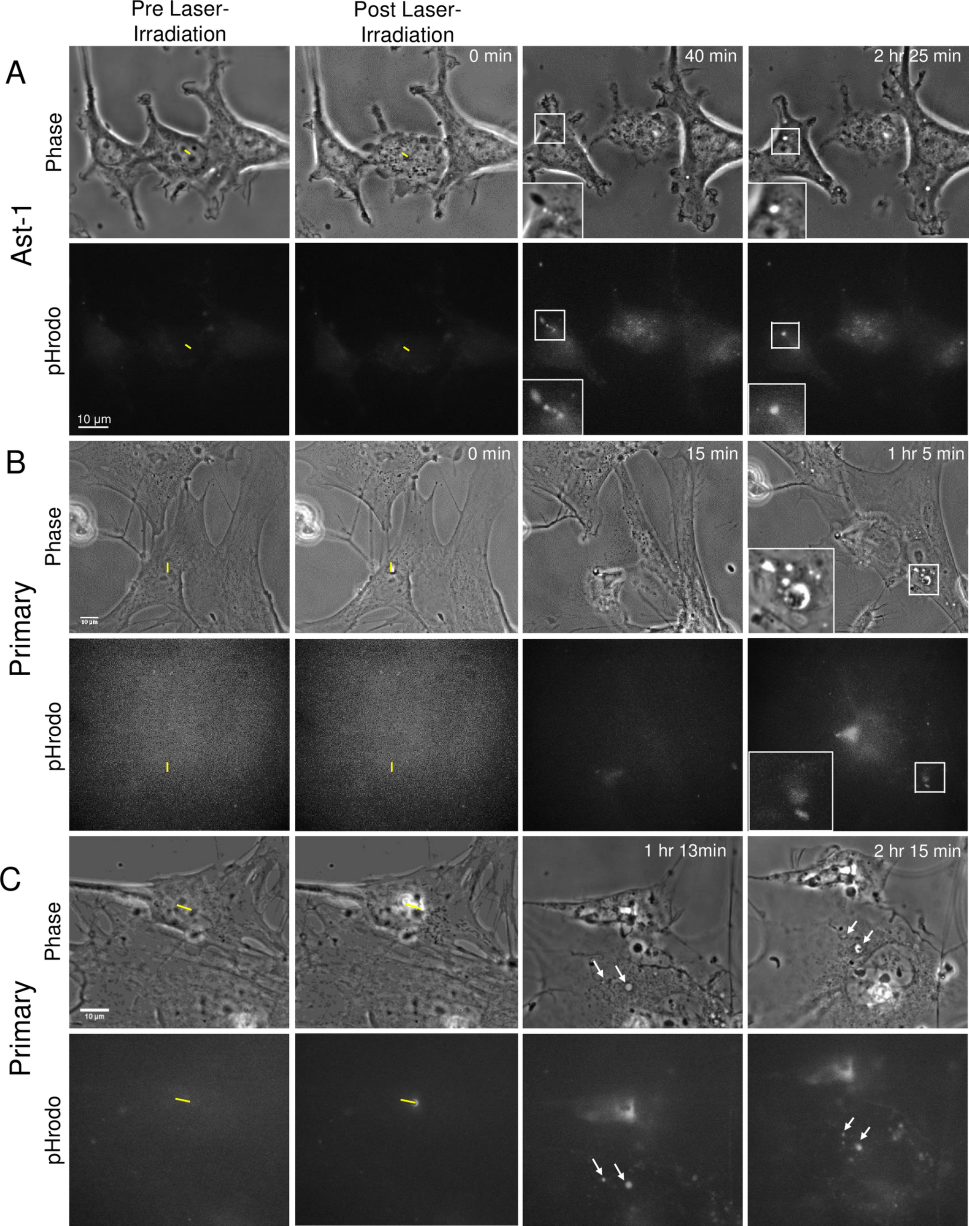
Formation of acidic endocytic vesicles in responding astrocytes. Phase contrast and corresponding fluorescence images of astrocytes from Ast-1 established astrocyte cell line (A) and primary cortical astrocytes (B and C) were irradiated in the presence of an intracellular pH indicator. Laser-irradiation resulting in cell death of the targeted cell (yellow ROI) shows no immediate change in intracellular fluorescence intensity of neighboring cells (Post Laser-Irradiation / 0 min images). A. At 40 minutes following laser irradiation endocytic vesicles being to form (magnified within the inset post-irradiation images). Corresponding fluorescence intensity increases at the position of newly formed endocytic vesicles confirm endosomes have an acidic pH. Multiple acidic vesicles visible at 40 minutes merge to form a larger acidic endosome, visible at 2 hr 25 minutes following laser irradiation. B and C. Formation of acidic vesicles in primary astrocytes. B. Inset highlights 8 endocytic vesicles, two of the largest near the bottom of the inset correspond to a concentrated fluorescence signal 1 hour and 5 minutes post laser irradiation. C. Arrows point to two large vesicles with corresponding pHrodo fluorescence visible between 1 hour 13 minutes and 2 hours 15 minutes post laser-irradiation.

The formation of acidic vesicles in response to laser irradiation of neighboring cells was observed in astrocytes from both the immortalized Ast-1 line and primary astrocytes (Fig 8A and 8B, respectively). Utilization of pHrodo intracellular pH indicator that is dimly fluorescent at neutral pH and intensely fluorescent at acidic pH allowed for the tracking of acidic vesicle formation. In pre-laser irradiation images, minimal fluorescence signal is visualized within the cytosol, denoting a neutral pH. Immediately following laser irradiation (post laser-irradiation/0 min images), no change in cytosolic pH is observed in astrocytes neighboring the targeted cell (yellow ROI). Over time, we observe vesicle formation via phase contrast imaging. Fluorescent images acquired confirm that some of the endocytic vesicles correspond to an increased fluorescence signal at vesicle positions (Fig 8). As the pH decreases within the vesicles, we observed an increase in fluorescence corresponding to the endosomes, suggesting that internalized debris is targeted for degradation within the acidic endosomes or lysosomes.

Similar to the immortalized astrocytes, primary astrocytes displayed an increased fluorescence at positions corresponding to multiple endocytic vesicles. Fig 8B and 8C depict the formation of multiple vesicles in pHrodo treated primary astrocytes responding to laser-irradiated neighboring cells. Vesicles visible in phase contrast images correspond to intense fluorescence signal in the two largest vesicles highlighted in the magnified insets of Fig 8B. Similarly two vesicles with corresponding fluorescence are visible at 1 hour and 13 minutes and 2 hours and 15 minutes post laser irradiation. The largest vesicles in both examples show visible cell debris residing within acidic vesicles in phase contrast images (Fig 8B at 1 hour 5 min image and 8C 2 hour 15 minute image).

### Isolated astrocytes migration toward damaged/dying cells

Analysis of the frequency of migration of a nearby astrocyte toward the cellular debris of a single killed cell suggested a distance-dependent relationship. Primary cortical cultures were plated at low density in order to permit observation of the interactions between isolated single astrocytes that did not share any membrane connection prior to laser-targeting of one of the cells. In 51/128 cases (40%), single isolated astrocytes migrated toward the irradiated cell and established a physical connection, referred to as “established contact” (Fig 2, two cells: 32 and 64 minutes post laser-irradiation). Newly formed connections varied from thin filopodial extensions from the astrocyte to the dying cell or its debris, to contact by the entire leading edge of the responding astrocyte. The latter situation often resulted in endocytosis of the cellular debris. The frequency of contact between the responding astrocyte and the necrotic cell/cell debris was determined by analysis of time-lapse images over a 60 to 90 minutes observation time following laser exposure (S2 Fig).

**Fig 9:**
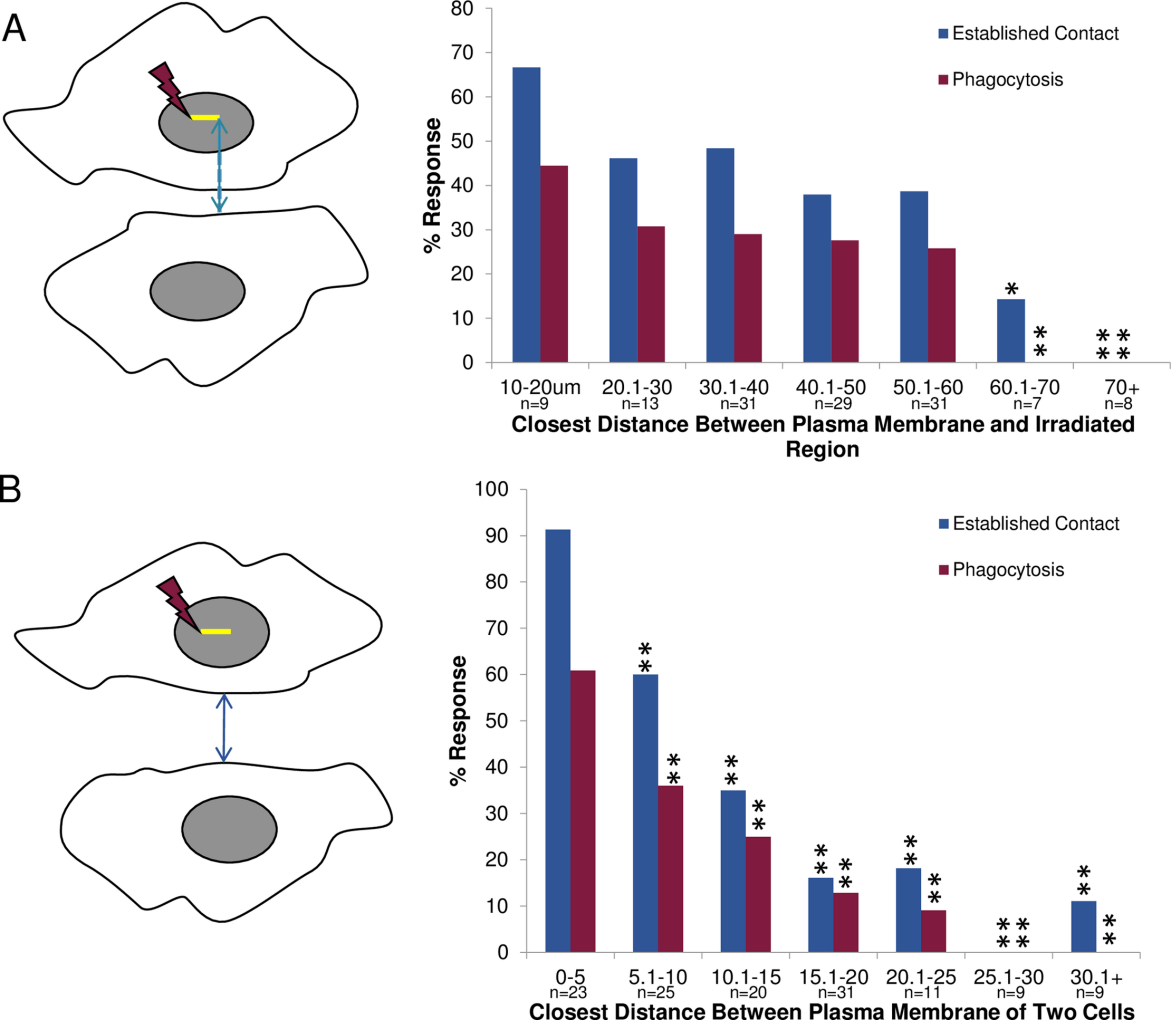
Distance dependent response of isolated astrocytes. **A. Response of cells based on the closest distance between the plasma membrane of the responding astrocyte and the laser-irradiated region.** A. Schematic diagram of distance measured between two observed cells. The blue line corresponds to the shortest distance between location of the laser ROI (yellow line) and the plasma membrane of the responding astrocyte. This is equivalent to the shortest distance material released from the irradiated region must diffuse to be sensed by the responding astrocyte. A higher rate of establishing cell-to-cell contact and subsequent phagocytosis was observed for cells situated closer to each other. A significant decrease in the responding astrocyte’s ability to establish contact with an irradiated cell occurred for cells separated by 60–70 μm (14%, p<0.05), and 70+ μm away (0%, p<0.01), as compared to 67% established contact response observed when the irradiated/lysed cell and the responding cell are 10–20 μm apart. Similarly, a significant decrease in phagocytic response was observed when the cells were 60–70 μm and 70+ μm microns apart (p<0.01) with a 0% phagocytic response, as compared to 44% observed for the 10–20 μm distance. **B. Response based on the closest distance between plasma membranes of the laser-irradiated cell and the responding cell**. C. Schematic diagram of the closest distance measured between the plasma membrane of two cells (blue line). As in Fig 9B, a higher rate of establishing cell-to-cell contact and phagocytosis was observed for cells closer to each other. A significant decrease (p<0.01) in the responding astrocyte’s ability to establish contact occurred for distances greater than 5 μm, when compared to those distance in the 0–5 μm range (91%).

The shortest distance (in μm) between the plasma membrane of the responding astrocyte and the point of laser exposure in the targeted cell was measured for each pair of isolated cells (Fig 9A). Prior to laser exposure, the closer the responding astrocyte was to the laser-targeted cell, the more frequently membrane contact between the two cells was established, and the more frequently phagocytosis resulted. For astrocytes separated by less than 40 μm prior to laser irradiation, 51% (27/53) established a local connection with the nearest adjacent cell; of these, 32% (17/53) underwent phagocytosis. A decreased response to establish contact, and subsequent phagocytosis, was found with increasing distance between the responding astrocyte and the laser ROI. At 10–20 μm separation, 6/9 (67%) established contact, of these, 4/9 (44%) underwent phagocytosis. At a distance of 20–30 μm 6/13 (46%) established contact and 4/13 (31%) of these initiated phagocytosis. At a distance of 30–40 μm, 15/31 (48%) established contact and 9/31 (29%) of these underwent phagocytosis. At 40–50 μm, 11/29 (38%) established contact, and of these 8/29 (28%) initiated phagocytosis. At 50–60 μm 12/31 (38%) established contact 8/31 (26%) of these underwent phagocytosis. For 60–70 μm separation only 1/7 (14%) established contact and this cell did not exhibit signs of phagocytosis. In one case, we observed an astrocyte migrate as far as 59 μm toward the laser-damaged necrotic cell and proceed to phagocytose it. Separation distances beyond 70 μm never resulted in an induced response from the nearby astrocytes (n = 8). Time-lapse videos of control astrocytes that were distant from the irradiated cell demonstrated that cells did not phagocytose non-injured cells (n = 74): thus, the phagocytic response is activated in response to damage to nearby cells.

In contrast to measuring the distance from the ROI to the irradiated cell, in this section we measured the minimum distance between the plasma membrane of the responding cell and the plasma membrane of the laser irradiated cell (Fig 9B). This analysis was undertaken to determine if the responding cell was sensing signals released from the damaged cell (not specific to the ROI targeted by the laser). Similar to the graph in Fig 9A, with increasing distance between the responding cell and the irradiated cell, there was a decrease in the initiation of contact and subsequent phagocytosis (Fig 9B). At the smallest cell-to-cell separation distance (0–5 μm), 21/23 (91%) established membrane contact, and of those 14/23 (61%) exhibited a phagocytic response. At greater distances both the establishment of contact, by either migration or extension of filopodial protrusions, and phagocytosis significantly decreased (p<0.01 for all distance ranges when compared to 0–5 μm): at 5–10 μm separation 15/25 (60%) established contact and 9/25 (36%) of these initiated phagocytosis, at 10–15 μm separation 7/20 (35%) established contact and of those 5/20 (25%) initiated phagocytosis, at 15–20 μm 5/31 (16%) established contact and of these 4/31 (13%) underwent phagocytosis, at 20–25 μm 2/11 (18%) established contact and of those 1/11 (9%) underwent phagocytosis. Beyond 25 μm separation, only one of eighteen cells established a visible membrane contact. No phagocytic events were initiated when cells were separated by a distance greater than 25 μm.

Isolated primary astrocytes migrated at an average rate of 0.5 μm/min while advancing on control, nontargeted neighboring cells. No significant increase in migration rate was observed for astrocytes responding to targeted cell debris when compared to random migration of astrocytes in culture, though there was a non-statistical mean increase to 0.66 μm/min (p = 0.22). Similarly for Ast-1 cells, we observed no significant difference between the average migration rate for nonirradiated Ast-1 control cells of 0.19 um/min and a rate of 0.12 um/min rate of Ast-1 cells responding to irradiated cell debris (p = 0.12).

### Membrane attachment effect on cell-cell interactions

**Fig 10:**
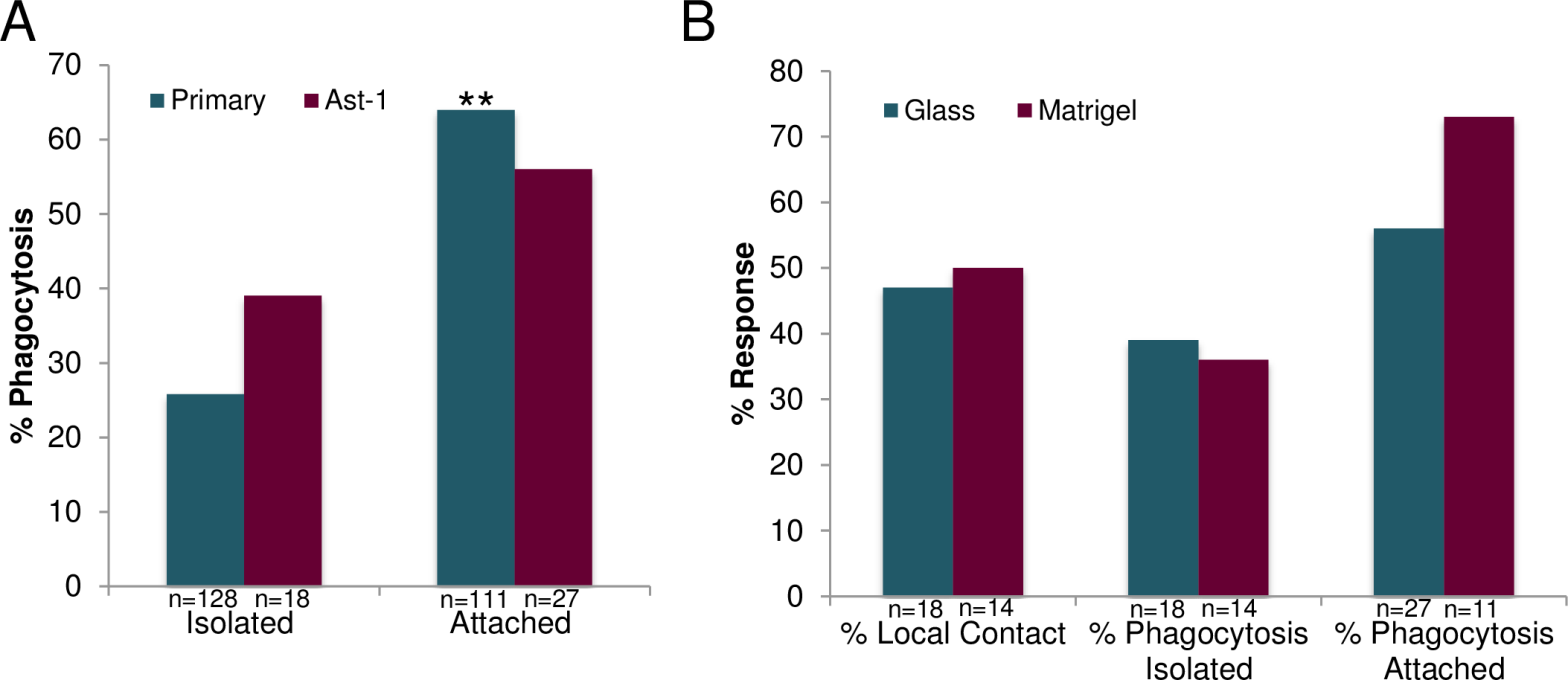
Increased phagocytic response for cells sharing a membranous connection. Isolated primary cells sharing no membrane connection had a significantly lower phagocytic response of 33/128 (26%), compared to 71/111 (64%) phagocytic responses for pairs of cells that shared a membrane connection (p<0.01). A similar decreased response was observed for astrocytes from the immortalized Ast-1 line. We observed a decrease in phagocytic response of isolated vs. attached cells with 39% phagocytic response of isolated Ast-1 vs. 56% phagocytic response of attached Ast-1 cells. However, this was not significant (p = 0.27). B. Ast-1 cells plated on two different substrates, glass or matrigel, were analyzed in response to laser-irradiation of neighboring cells. Response of single, isolated cells was very similar on glass and matrigel substrates, for both local contact (47% glass and 50% matrigel) and phagocytosis (39% glass and 36% matrigel). Attached astrocytes had a higher frequency of induction of phagocytosis when plated on matrigel at 73% vs. plated on glass 56%, however this was not a significant increase (p = 0.33).

To determine the importance of astrocyte cell-to-cell interactions in phagocytosis, we compared the response of single isolated cells to cells within multicellular clusters. Each cell in a cluster was in contact with at least one other cell in the cluster (Fig 10). We found a significant difference in the frequency of phagocytic response between single isolated primary astrocytes with no membrane attachment to the targeted cell, and groups of primary astrocytes in a multicellular cluster (referred to as “attached cells”) (Fig 10). The results indicate that an astrocyte in a group of connected cells was significantly more likely to phagocytose the remnants of an irradiated cell (killed) in the group (64%, 71/111), than a single isolated astrocyte was to phagocytose another single irradiated (killed) astrocyte (26%, 33/128; p<0.01). The average separation between isolated primary astrocytes and the ROI of isolated irradiated astrocytes was 43.7 μm. Connected astrocytes were separated from the ROI by an average distance of 30.2 μm.

A similar response was observed between immortalized Ast-1 astrocytes and those isolated from primary cortical tissue (Fig 10). Following laser irradiation, 7/18 (39%) isolated Ast-1 cells induced a phagocytic response, similar to the 26% response (33/128) observed for isolated primary astrocytes. In the case of attached Ast-1 cells, 15/27 (56%) cells induced phagocytosis in comparison to 64% (71/111) phagocytic rate observed in attached primary astrocytes. In comparison with primary astrocytes no significant difference was observed between astrocytes derived from primary cortical tissue vs. immortalized Ast-1 line (p = 0.24 isolated, p = 0.42 attached).

To determine if the substrate affects the phagocytic response of astrocytes in vitro, cells from the immortalized Ast-1 line were plated on both glass and matrigel-coated glass. The response rate for both local contact and phagocytosis were analyzed for single isolated cells (Fig 10). No significant difference was observed in Ast-1 cells establishing local contact between isolated cells plated on glass 8/18 (47%) or matrigel 7/14 (50%), p = 0.76. Additionally no significant difference was observed in the phagocytic response of primary isolated astrocytes plated on glass 7/18 (39%) or matrigel 5/14 (36%), p = 0.86. Although attached Ast-1 cells plated on matrigel were more likely to induce phagocytosis 73% (8/11) vs. 56% (15/27) attached cells plated on glass, this response was not significant, p = 0.33. The stiffness of the substrate did not significantly affect the response of astrocytes to laser-damaged neighboring cells.

**Fig 11:**
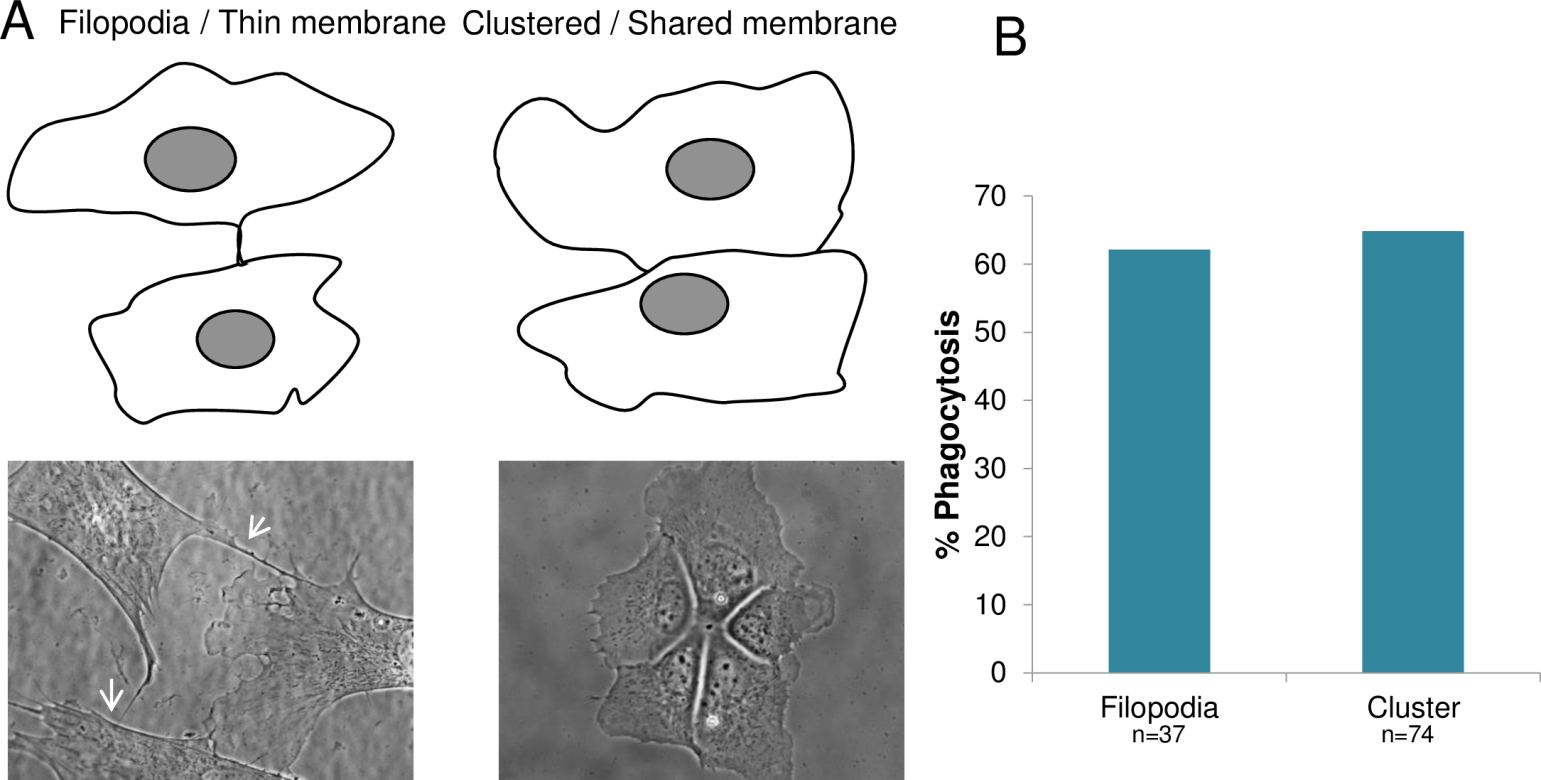
Amount of shared membrane does not affect the phagocytic response of astrocytes. A. Cells were categorized by the amount of membrane connection shared between two astrocytes. Two different connections between cells were observed: those with thin filopodial connections “Filopodia/ thin membrane”, and clusters of cells with large amount of shared membrane, “Clustered/ shared membrane”. Phase contrast images (contrast enhanced) show representative cells below the schematic diagram of the two categories of observed membrane connections. White arrows show thin filopodial connections, while significantly more shared membrane between cells is visible in the cell cluster on the right. B. No significant difference in phagocytosis was observed between cells sharing thin filopodial connections versus cells in clusters with significant shared membrane: 62% (23/37) and 65% (48/74), respectively.

To determine if the amount of shared membrane between connected cells affected the ability to induce phagocytosis, connected cells were divided into two categories: (1) cells connected by thin filopodial extensions (Fig 11 filopodia/ thin membrane), and (2) clusters of two or more cells with extensive membrane contact (Fig 11, clustered/ shared membrane). Filopodial connections are defined as having a cytoplasmic width below 1.5 μm, (mean = 0.9 μm). Clusters of cells had a mean of 27.3 μm of shared membrane surface. Surprisingly, cells that shared a large membranous area had no significant increase in phagocytic response compared to astrocytes that shared thin filopodial connections (p = 0.78): 65% (48/74) of cells in clusters initiated phagocytosis, while cells with only a thin filopodial connections to the targeted cell initiated phagocytosis with a frequency of 62% (23/37) (Fig 11). On average astrocytes joined by filopodia were separated from the ROI by 40.3 μm, and clustered cells were separated from the ROI by 25 μm.

### Phagocytosis of neurons by astrocytes (astrocyte-neuron)

**Fig 12:**
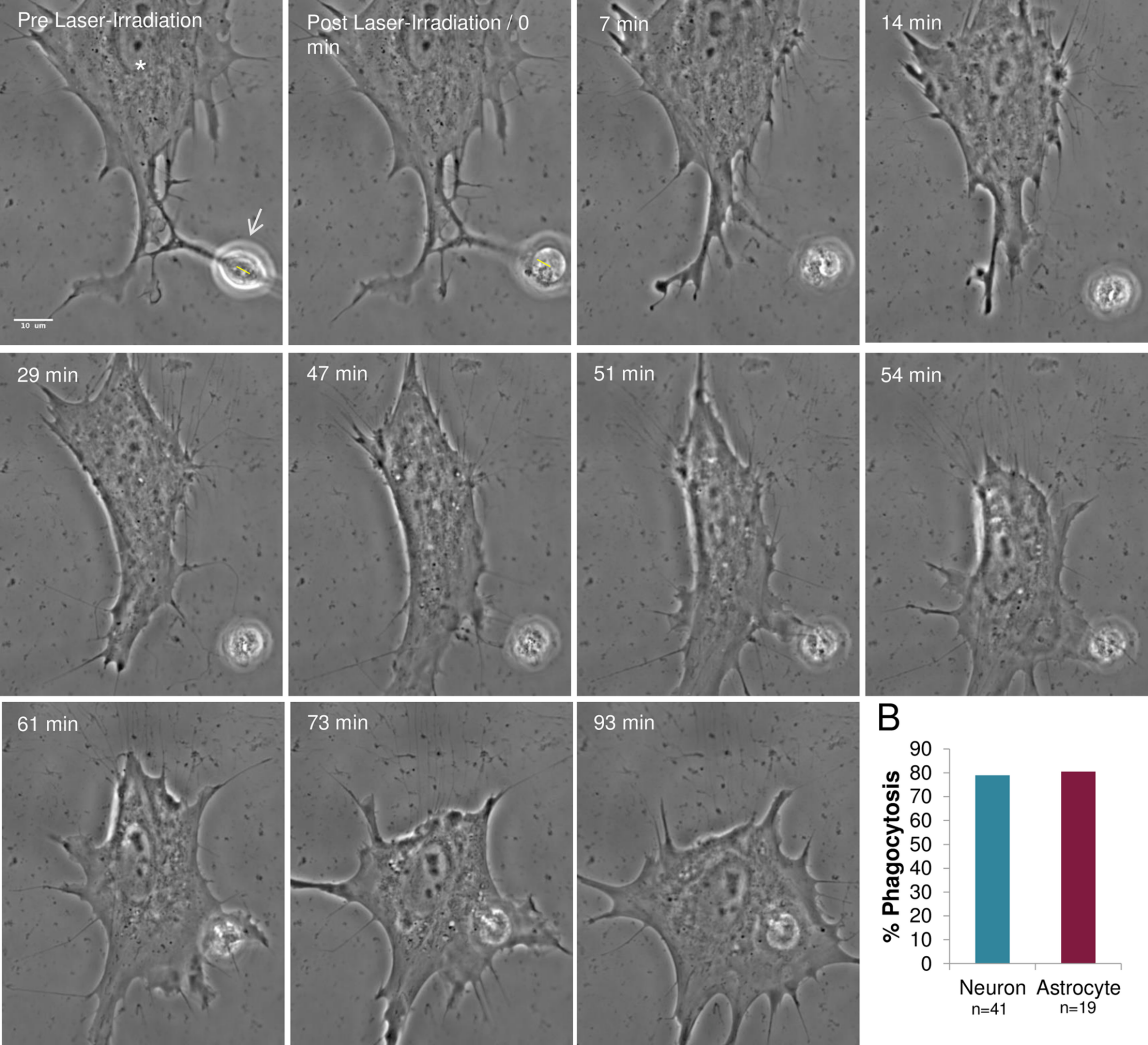
Phagocytosis of laser-irradiated neurons by neighboring astrocytes. A. Neurons from mixed primary cortical cultures that shared membrane contact with neighboring astrocytes were targeted in the nucleus (yellow ROI). Neurons damaged by laser exposure rounded and released significant cell debris as visualized in the Post Laser-Irradiation image. The responding astrocyte (white asterisk) engulfed the cellular debris 56–75 minutes following irradiation. This response is similar to phagocytosis of laser-irradiated astrocytes. B. Astrocyte responses to laser-killed neurons are similar to responses to laser-killed astrocytes. Results are similar to astrocytes sharing a membrane connection, with rates of phagocytosis of lysed astrocytes at 81% (15/19), and lysed neurons at 79% (32/41).

To determine whether the debris from laser-irradiated neurons can stimulate astrocyte phagocytosis, the nucleus of individual neurons with a membrane connection to a neighboring astrocyte was targeted with the laser (Fig 12, yellow ROI in Pre Laser-Irradiation and Post Laser-Irradiation images). Since the nucleus occupies the majority of the cell body, it was difficult to lyse the neuron via cytoplasmic irradiation. Therefore, in all of the neuronal irradiation experiments, cell lysis/death was performed via irradiation of the nucleus. Rapid cell death was observed following laser-irradiation as evidenced by dramatic rounding of the neuron (Fig 12, S7 Fig). Nearby connected astrocytes responded by migrating toward, and phagocytosing the neuronal debris. The response occurred with a frequency of 79% (15/19). This was similar (p = 0.59) to astrocyte-astrocyte phagocytosis previously described.

Cell morphology changes in the responding astrocyte, including the formation of endocytic vesicles in astrocytes reacting to damaged or killed neurons were similar to those observed in the astrocyte-astrocyte experiments. There was no significant difference in the distance separating the ROI and the responding cell between astrocytes-astrocyte studies (average 30.2 μm) and neuron-astrocyte studies (average 25.6 μm, p = 0.76 via t test), (Fig 9A).

### Effect of subcellular targeting site

**Fig 13:**
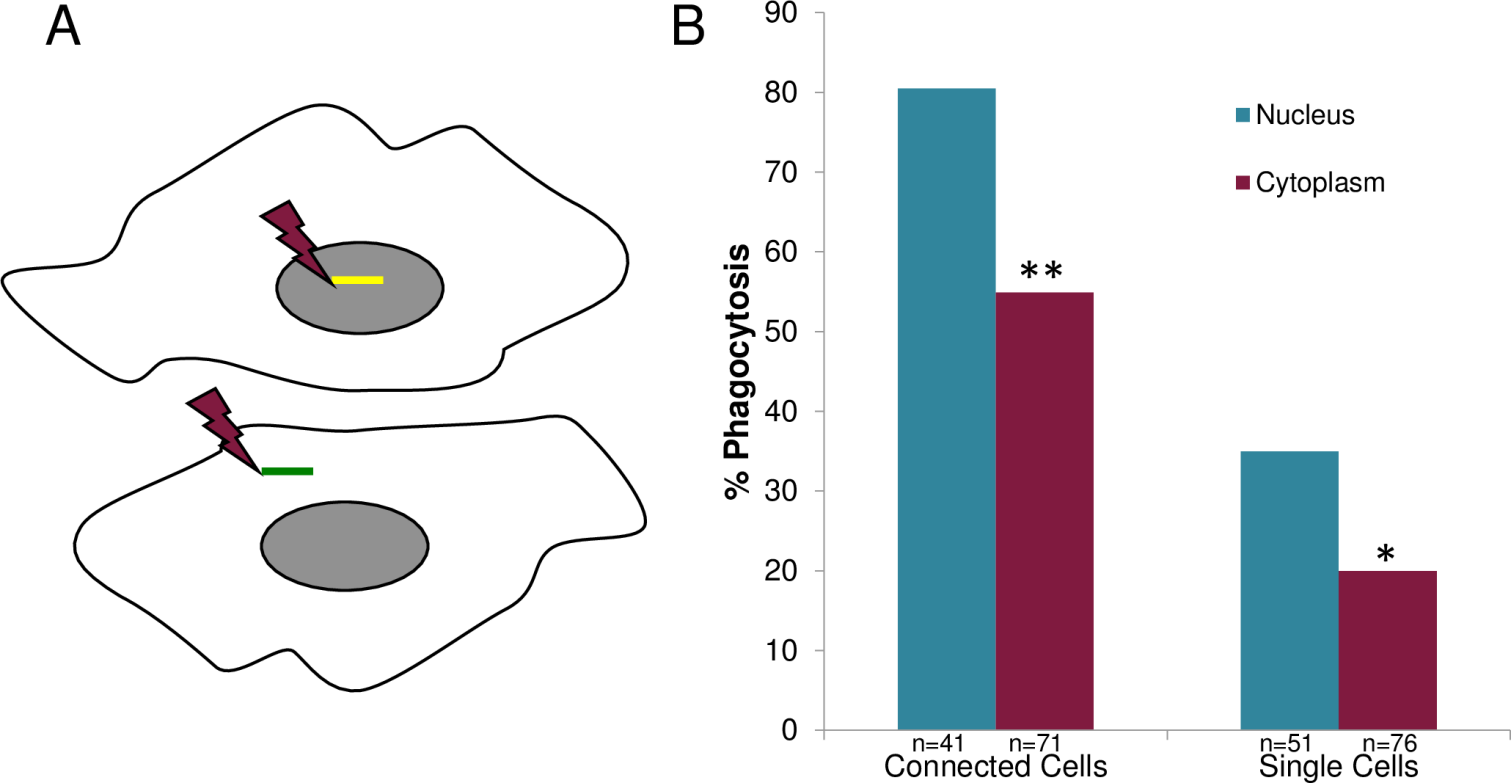
Nucleus targeted irradiation results in a higher phagocytic response than cytoplasm targeted irradiation. A. Schematic diagram depicting position of laser irradiation for nucleus targeted and cytoplasm targeted cells. B. A significant decreased in phagocytic response for cytoplasm-irradiated cells vs. nucleus-irradiated cells was observed for two categories: (1) cells with a membrane connection, and (2) isolated cells without a membrane connection. For connected cells, 80% (33/41) phagocytic response for nucleus-targeted astrocytes vs. 55% (39/71) for cytoplasm-targeted astrocytes was observed (**p>0.01). For isolated non-membrane-connected cells, we observed 35% (18/51) phagocytic response for nucleus-targeted cells versus a 20% (15/76) phagocytic response for cytoplasm-irradiated astrocytes (*p = 0.05).

To determine whether the subcellular targeting site affects the phagocytic response of astrocytes, cells were killed by either nuclear or cytoplasmic targeting (Fig 13A). In both nuclear and cytoplasmic laser-irradiation, rapid cell death was confirmed by PI staining (S6 Fig). Analysis of the neighboring astrocytes’ response revealed a significantly higher phagocytic response when the target cell was killed by nuclear as opposed to cytoplasmic irradiation (Fig 13B). This was observed for both membrane-connected and isolated cells. For connected cells, 80% (33/41) of nucleus-irradiated astrocytes induced phagocytosis versus 55% (39/71) for cytoplasm-irradiated cells (p<0.01). For isolated single cells, 35% (18/51) of nucleus-irradiated astrocytes induced phagocytosis versus 20% (15/76) of cytoplasm-irradiated astrocytes (p = 0.05).

## Discussion

In this study we demonstrate that a single laser damaged or killed astrocyte or neuron induces a nearby astrocyte to initiate phagocytosis. The ability to observe the response to a single damaged cell demonstrates the efficiency of the nervous system to sense damage. The dramatic observation that damage to an individual cell, ranging from the dissection of a neuronal process to the lysis of cell, is enough to trigger the “clean-up” response. These observations provide novel functional information about the phagocytic process, which is essential for nervous tissue function. Visual observation of phagocytosis by time lapse imaging is confirmed by the following observations: (1) growth cone engulfment, (2) the engulfment of PI-labeled cellular debris into vesicles, and (3) the formation of acidic endocytic vesicles including visible debris within these vesicles. If unattached to the irradiated cell, the responding astrocyte first migrates toward, and subsequently phagocytoses the debris of the irradiated cell. If the responding astrocyte is in contact with the target cell prior to laser irradiation (either by thin filamentous filopodia or by a substantial area of common membrane), the responding astrocyte initiates phagocytosis soon after the target cell is irradiated. Our results demonstrate that astrocytes limit the spread of injury to surrounding regions in the CNS, a function similar to microglia. Microglia have been shown to respond to extracellular nucleotides released by damaged cells, leading to phagocytosis and degradation of apoptotic cells [11]. Despite the fact that astrocytes play a significant role in the maintenance of CNS homeostasis, few studies have documented the details of astrocyte phagocytosis, and none have done this at the single cell level.

Cellular morphology changes in response to debris from laser-irradiated cells varied from no response, to formation of filopodia or lamellipodia. This was observed by time lapse imaging and by differences between rates of migration to establish contact, and by initiation of phagocytosis. Variation in the reaction of responding astrocytes could be dependent on the RhoGTPase members such as Cdc42, which is known to induce formation of filopodia [12]. Additionally, Cdc42 has the potential to stimulate Rac, which induces lamellipodia formation [12]. Cells that were observed to establish connections but were not able to sustain the connection, did so with filopodial connections and did not progress with a phagocytic response. This would be consistent with the activation of Cdc42 but not Rac. For those cases where phagocytosis occurred, the responding astrocyte engulfed debris with an advancing lamellipodium. In these cases, activation of Rac could result in a higher degree of response to cell lysis. The ability to induce phagocytosis and observe cells at high resolution will facilitate future studies on signal transduction and the control of astrocyte’s phagocytic response. The addition of activators and inhibitors should stimulate astrocyte phagocytic response to damaged or dead cells.

One previous study recorded astrocytes phagocytosing cellular debris after an extensive region of damage was made by moving a scalpel blade through a mixed monolayer culture containing neurons, oligodendrocytes and astrocytes [6]. In that study, each culture received 20 cuts, 2 mm apart. Cells of numerous types were indiscriminately damaged and/or killed, with a resulting release of large amounts of nuclear and cytoplasmic material. Our studies differ in that they are on specific selected single cells (either an astrocyte or a neuron). We demonstrate that the selective damage and/or lysis of just a single astrocyte or neuron is sufficient to induce phagocytic reactivity in a neighboring astrocyte.

The precision of short-pulsed laser nanosurgery allows subcellular and cell-specific damage to selected cells rather than indiscriminant damage to a large number of cells of a mixed cell type, such as neurons, astrocytes, and microglia, as described previously [6]. This permits investigation of a single astrocyte’s ability to respond to damage of an adjacent cell, and to determine if this response is cell-type specific. We saw no difference in response to cell debris of neurons versus astrocytes. The results further demonstrate that a signal (or signals) released by one cell is sufficient to cause a profound effect (phagocytosis) in another cell. Our results demonstrate that there is a physical distance threshold of approximately 60 μm for an astrocyte to respond to a dead cell or its debris. It is likely that the threshold for the phagocytic response is related to the decrease in concentration of diffusing molecules as a function of the distance between receptors on the responding astrocyte and the dead/dying cell. The differential response of phagocytosis based on the distance separating the laser-targeted cell and an unattached responding astrocyte is a novel observation that should be further pursued in future studies. Studies on molecular signaling, membrane receptor response, and internal signal transduction are now possible at the single cell level.

As mentioned, the results reported here demonstrate that the release of cellular material from a single lysed cell is adequate to cause a neighboring astrocyte to react. The responding astrocyte migrates toward the damaged cell (debris), initiates physical contact, and often phagocytosis. Vernon and Tang [13] analyzed the mechanism that cells used to remove dying cells. If the injured cells were not removed, neighboring healthy cells died as well. The damage-associated molecular pattern (DAMP) includes molecules such as calcium, ATP and DNA. The DAMP molecules are thought to be the molecular triggers for macropinocytosis and phagocytosis. The formation of large vesicles we observed in astrocytes responding to the laser-lysed cellular debris is consistent with these processes. Our results showing a higher incidence of phagocytosis in a responding astrocyte when the nucleus of the target cell was irradiated, suggests that DNA (one of the DAMP molecules) or other possible nuclear factors can enhance the phagocytic response.

The difference observed in astrocyte response to cellular debris when cells are in contact with each other in a cluster versus single isolated cells may suggest a mechotransduction control component of the phagocytic response. Retraction that results in a pulling force of the targeted cell on the responding cell could cause an increase in tension on the membrane of the reacting astrocyte. A single thin filopodium could be sufficient to physically sense the injury and induce the same response as a significantly larger membrane area shared between the lysed cell and the responding astrocyte. This suggests that astrocytes might be able to respond to changes in tension resulting from damage to neighboring cells. This result is relevant to *in situ* situations where cells often exist in interconnecting networks. There is growing evidence for the involvement of mechanical effects in the reactivity of astrocytes to traumatic brain injury [5]. The importance of the intermediate filament network and integrins to activate mechanosensitive channels, including calcium channels, to induce astrocyte reactivity can now be investigated in the single/multi-cellular systems we describe.

## Conclusions

In summary, we demonstrate the ability of two short-pulsed (nanosecond and femtosecond) laser systems to successfully damage or lyse single neurons and astrocytes. In response, non-irradiated nearby astrocytes migrate toward, and phagocytose the dead and/or dying cells. We demonstrate that a membrane connection between the astrocyte and the targeted cell (another astrocyte or a neuron) prior to laser-targeting, results in a higher rate of phagocytosis. We further demonstrate that there is adequate signal from a dissected neurite and single damaged/killed cells to activate the phagocytic response of astrocytes as far away as 60 microns. The nature of this signal(s) and the reactive response in the activated astrocyte, are yet to be determined. The ability to initiate a phagocytic response at the single or multicellular level provides an *in vitro* system to study the basic mechanisms of astrocyte phagocytosis in the nervous system. The ability to induce astrocyte phagocytosis in single and multicellular environments allows further study of an important role of astrocytes in maintaining homeostasis.

## Supporting information

S1 Fig Phagocytic response of established Ast-I cells. Time lapse video of immortalized astrocyte type-I cell phagocytic response to laser-irradiated neighboring astrocytes.
(M4V)

S2 Fig Isolated astrocyte response to laser induced cell lysis. Time lapse video demonstrates the dynamic phagocytic response of primary astrocytes with no membrane connection to laser-lysis of a target cell.
(M4V)

S3 Fig Astrocyte response to laser induced cell lysis in a multicellular cluster. Time lapse video demonstrates the dynamic phagocytic response of astrocytes in a multicellular cluster to laser-lysis of another astrocyte within the same cluster.
(M4V)

S4 Fig Vesicle formation and engulfment of laser-irradiated cellular debris. Time lapse video demonstrating dynamic endocytic vesicle formation as responding astrocyte engulfs cellular debris.
(AVI)

S5 Fig Astrocyte response to a laser dissected neuron process. Responding astrocyte fully engulfs the remaining active growth cone, including endocytic vesicle formation. As the astrocyte crawls away, no visible debris remains of the dissected growth cone.
(AVI)

S6 Fig Propidium iodide inclusion confirms laser induced cell death. Prior to laser exposure, propidium iodide (PI) is prevented from entering the cell due to intact membrane integrity. Immediately following laser irradiation (irradiated cells shown with yellow ROI), PI enters the irradiated cell and intercalates into the DNA of the dead cell. Inclusion of PI is detected as an increase in fluorescence in both the nucleus (A) and cytoplasm (B) of the targeted cell. No increase in fluorescence is observed within non-targeted neighboring cells following laser irradiation. A similar increase in fluorescence is detected in a laser-irradiated neuron (C).
(TIFF)

S7 Fig Astrocyte response to laser targeted cell body of a neuron. Time lapse video demonstrates the dynamic phagocytic response of an astrocytes to a laser-irradiated neuron.
(M4V)
