# Supplementary figures and images for: Phagocytic response of astrocytes to damaged neighboring cells
Source: PLoS One. 2018 Apr 30;13(4):e0196153. doi: 10.1371/journal.pone.0196153 (PMC5927416; doi:10.1371/journal.pone.0196153)

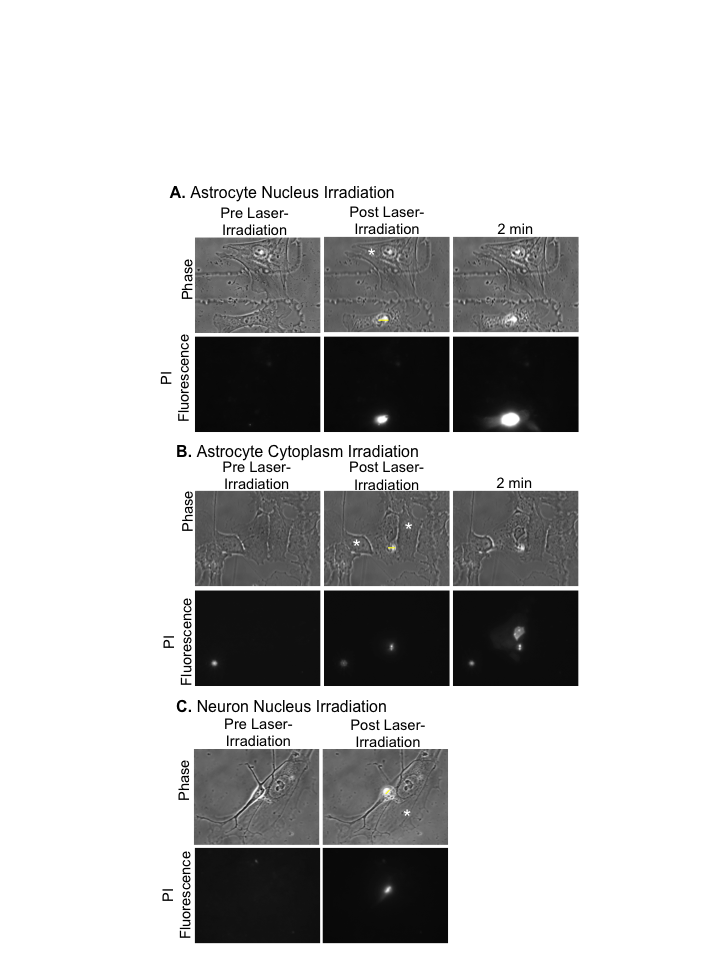

Supplement: S6 Fig — Prior to laser exposure, propidium iodide (PI) is prevented from entering the cell due to intact membrane integrity. Immediately following laser irradiation (irradiated cells shown with yellow ROI), PI enters the irradiated cell and intercalates into the DNA of the dead cell. Inclusion of PI is detected as an increase in fluorescence in both the nucleus (A) and cytoplasm (B) of the targeted cell. No increase in fluorescence is observed within non-targeted neighboring cells following laser irradiation. A similar increase in fluorescence is detected in a laser-irradiated neuron (C). (TIFF) [file pone.0196153.s006.tiff]
